# ‘Putting Life in Years’ (PLINY) telephone friendship groups research study: pilot randomised controlled trial

**DOI:** 10.1186/1745-6215-15-141

**Published:** 2014-04-24

**Authors:** Gail A Mountain, Daniel Hind, Rebecca Gossage-Worrall, Stephen J Walters, Rosie Duncan, Louise Newbould, Saleema Rex, Carys Jones, Ann Bowling, Mima Cattan, Angela Cairns, Cindy Cooper, Rhiannon Tudor Edwards, Elizabeth C Goyder

**Affiliations:** 1 ScHARR, University of Sheffield, Regent Court, 30 Regent Street, Sheffield S1 4DA, UK; 2 Clinical Trials Research Unit, ScHARR, University of Sheffield, Regent Court, 30 Regent Street, Sheffield S1 4DA, UK; 3 Institute of Medical and Social Care Research, Bangor University, Bangor, Gwynedd LL57 2DG, UK; 4 School of Health Sciences, Highfield Campus, University Road, Southampton SO17 1BJ, UK; 5 Faculty of Health and Life Sciences, University of Northumbria, Coach Lane Campus, Newcastle Upon Tyne NE7 7XA, UK; 6 Community Network, First Floor, 12-20 Baron Street, London N1 9LL, UK

## Abstract

**Background:**

Loneliness in older people is associated with poor health-related quality of life (HRQoL). We undertook a parallel-group randomised controlled trial to evaluate the effectiveness and cost-effectiveness of telephone befriending for the maintenance of HRQoL in older people. An internal pilot tested the feasibility of the trial and intervention.

**Methods:**

Participants aged >74 years, with good cognitive function, living independently in one UK city were recruited through general practices and other sources, then randomised to: (1) 6 weeks of short one-to-one telephone calls, followed by 12 weeks of group telephone calls with up to six participants, led by a trained volunteer facilitator; or (2) a control group. The main trial required the recruitment of 248 participants in a 1-year accrual window, of whom 124 were to receive telephone befriending. The pilot specified three success criteria which had to be met in order to progress the main trial to completion: recruitment of 68 participants in 95 days; retention of 80% participants at 6 months; successful delivery of telephone befriending by local franchise of national charity. The primary clinical outcome was the Short Form (36) Health Instrument (SF-36) Mental Health (MH) dimension score collected by telephone 6 months following randomisation.

**Results:**

We informed 9,579 older people about the study. Seventy consenting participants were randomised to the pilot in 95 days, with 56 (80%) providing valid primary outcome data (26 intervention, 30 control). Twenty-four participants randomly allocated to the research arm actually received telephone befriending due to poor recruitment and retention of volunteer facilitators. The trial was closed early as a result. The mean 6-month SF-36 MH scores were 78 (SD 18) and 71 (SD 21) for the intervention and control groups, respectively (mean difference, 7; 95% CI, -3 to 16).

**Conclusions:**

Recruitment and retention of participants to a definitive trial with a recruitment window of 1 year is feasible. For the voluntary sector to recruit sufficient volunteers to match demand for telephone befriending created by trial recruitment would require the study to be run in more than one major population centre, and/or involve dedicated management of volunteers.

**Trial registration:**

ISRCTN28645428.

## Background

Many older people live in social isolation, with those over the age of 65 and living in the UK twice as likely as other age groups to spend over 21 hours of the day alone [1]. Social isolation and loneliness are strongly correlated with poor physical and mental health outcomes and admission to residential care [2], while low levels of social engagement in older people are related to morbidity and mortality [3]. On the other hand, strong social networks are known to be protective of physical and mental health [4-6]. As a result, policy makers are encouraging the delivery of health promotion interventions to older people with the aim of compressing morbidity and increasing quality of life in the later years of life [7-12].

In 2008, the UK National Institute for Health and Clinical Excellence (NICE) highlighted shortcomings in the evidence (identified by systematic reviews) for interventions that promote mental wellbeing in older people [11-13]. In particular, one review suggested that the most effective interventions were those conducted in a group with educational and/or supportive input [13]. As a result, the PLINY study was commissioned to establish whether a home-based intervention could improve or successfully maintain the mental wellbeing of older people living in the community with a focus upon those who are vulnerable and hard to reach. Qualitative research reported since the publication of the NICE guidance suggested that telephone befriending services promoted by two UK national charities “helped older people to gain confidence, re-engage with the community and become socially active” [14]. The authors’ recommendation, that initial one-to-one telephone calls with older people might be used to encourage participation in telephone clubs [15], echoed that of an earlier randomised controlled trial (RCT) conducted in the US [16]. It was these research findings which informed the design of the PLINY intervention, the evaluation of which we report here.

We undertook an RCT to evaluate the effectiveness and cost-effectiveness of a telephone befriending intervention compared with usual health and social care provision for the maintenance of health-related quality of life (HRQoL) in community-based older people. Telephone befriending is an example of a complex intervention, in that it has several interacting components [17]. As evaluation of complex interventions is challenging [17], and publicly funded trials often fail due to inadequate participant recruitment [18]; the UK National Institute for Health Research tend to commission RCTs with internal pilots to assess feasibility of the full or main study. In this paper, we report the results of the internal pilot trial and discuss the decision to stop the main trial early due to feasibility concerns. The dissemination of pilot trial results to inform the design and conduct of future studies is widely recommended [19,20]. The results of our pilot study have utility, beyond underpinning further trials of telephone befriending, as they incorporate rarely documented issues with the delivery of health and social care interventions by the voluntary sector. This pilot trial does not attempt to provide evidence for the clinical effectiveness of telephone befriending for the maintenance of HRQoL in older people. For this reason, our findings are presented using the CONSORT 2010 checklist [21] (Additional file 1), but are also in conformity with proposed CONSORT-modifications for reporting the results of pilot studies and pragmatic trials [22,23].

## Methods

The protocol for the main trial, including the internal pilot, is available from the National Institute for Health Research (NIHR) website [24].

### Trial design

This paper reports on the internal pilot trial of a parallel-group RCT with a 1:1 allocation ratio.

### Participants and setting

Between June 2011 and December 2013, eighteen general practices sent brief study information and invitations to contact the research team to 9,051 people aged 75 years and over from their practice lists. Further invitations were sent to 528 participants of an existing longitudinal observational study who had consented to be contacted about further research [25]. A total of 2,000 recruitment packs containing similar invitations were issued to local NHS, social care and third sector organisations who agreed to distribute them; the number subsequently received by the target population is unknown.

Research assistants posted the participant information sheet to those who expressed an interest, and then telephoned respondents to arrange a screening visit, which was held in their own home. Those eligible for the study: (a) were aged 75 or over; (b) had good cognitive function, defined as Six Cognitive Impairment Test (6CIT [26]) score of 7 or under; (c) lived independently (alone or with others) or in sheltered housing; and (d) could converse in English. We excluded those who: (a) could not use a telephone even if provided with appropriate assistive technology; (b) lived in residential/nursing care homes; and (c) were already receiving telephone interventions. Those scoring 7 or more on the 6CIT were contacted by a clinically qualified member of the research team, informed of their score and advised to contact their general practitioner, and excluded from the study.

### Interventions

After confirmation of eligibility and consent, we used a centralised web based randomisation service provided by the Sheffield Clinical Trials Research Unit central web-based randomisation service to allocate participants to either: (1) telephone befriending group intervention; or (2) a control group receiving no treatment as part of the study protocol. Participants in both groups continued to receive usual health and social care outside of the study protocol. The principal investigator and study statisticians were blinded to treatment allocation codes until the final analysis was complete.

The aim of the group intervention was to help older people maintain good mental health by increasing the extent of their social networks. The group intervention was preceded by using one-to-one telephone befriending to encourage participants to join telephone friendship groups [14]. The intervention was delivered by volunteers, with no previous experience of one-to-on befriending or group facilitation, who were recruited by a local franchise of a national UK charity dedicated to improving the lives of older people (Age UK), hereafter ‘the provider’. Following delivery of a standard induction programme for new volunteers, the provider’s Customer Engagement Manager then facilitated training in one-to-one befriending to groups of between two and seven volunteers in one session lasting between 1 and 2.5 hours. This initial training covered topics consisting of issues of confidentiality and equality, information about the research study, and training in making one-to-one calls. Training in group facilitation was then delivered to all volunteers by the same professional trainer who was experienced in this role. This training used standardised content developed for telephone friendship group facilitators working with Community Network, a national charity and social enterprise which supplies a range of commercial telephone services to the third sector. Up to five volunteers simultaneously received four 1-hour training sessions in group facilitation skills, delivered over Community Network’s teleconferencing system. Training involved using scenarios to teach volunteers how to facilitate cohesive groups thereby providing a safe and supportive environment for achieving group goals. It also entailed skills development to be able to intervene in the event of conflict or where the pre-defined participant ground-rules were broken [27]. The training was supported by a written manual for the volunteer befrienders. Community Network and the service provider harmonised their policies on confidentiality and safeguarding ‘clients’ for the purposes of study intervention delivery. To avoid cost to the volunteers and participants, one-to-one and group calls were made through the Community Network teleconferencing system and paid for by Age UK (National).

Initial one-to-one befriending involved 10- to 20-minute calls once per week for up to 6 weeks made by the volunteer befriender to an allocated participant. One-to-one calls aimed to familiarise the participant with the volunteer, conduct everyday conversation and prepare participants for the telephone friendship groups.

Subsequent friendship groups consisted of up to six participants and involved 1 hour teleconferences, at a pre-arranged time, once per week for 12 weeks facilitated by the same volunteer as had conducted one-to-one befriending. Friendship groups did not aim to induce behaviour change but to reduce social isolation by providing a safe environment for building relationships, sharing experiences, companionship and support, thereby maintaining participants’ sense of confidence and mental well-being.

### Clinical outcomes

The primary clinical endpoint was the level of mental wellbeing at 6-months post-randomisation, measured using the Short Form (36) Health Instrument (SF-36) mental health (MH) dimension [28]. Secondary endpoints were: other dimensions of the SF-36 to measure functional health and well-being; subjective wellbeing, measured using the Office for National Statistics (ONS) approach [29]; health status, measured with the EuroQol 5-Dimension (EQ-5D) questionnaire [30]; symptoms of self-reported depression, using the Patient Health Questionnaire (PHQ-9) [31]; optimistic self-beliefs about the ability to cope with difficult life using the 10-item General Perceived Self Efficacy (GSE) scale [32]; overall, emotional, and social loneliness, using the 11-item De Jong Gierveld Loneliness Scale [33]; and a bespoke health and social care resource use questionnaire. A cost-effectiveness analysis was planned but, due to early closure of the main trial, was not undertaken [25]. In the absence of data on actual expenditure, a detailed breakdown of the provider and trainer budgets will appear in the NIHR monograph.

### Sample size for the main trial

The SF-MH dimension is scored on a 0 (poor) to 100 (good health) scale, with between-group differences of between 5 and 10 points considered “clinically and socially relevant” [34]. In a general population survey of 3,084 community residents, the mean SF-36 MH score was 68.3 with an SD of 19.9 [35]. We assumed a correlation of 0.50 between the baseline and 6-month MH score, an average cluster size of six participants per telephone befriending group and an intra-cluster correlation (ICC) of 0.04, with a design effect of 1.28. With 80% power to detect a mean difference of eight points in 6-month MH scores and allowing for 20% loss to follow-up, the main trial required recruitment and randomisation of 124 people per arm (248 in total).

### Feasibility criteria

The internal pilot study assessed two formal progression criteria. In order to continue the full trial to completion the trial team had to: (1) recruit a minimum of 68 participants in 95 days; and (2) collect valid primary outcome data for 56 (80%) of those recruited 6 months later. A third progression criterion, which was not defined in formal quantitative terms in the research protocol, was that the service provider should be able to recruit, train and retain enough volunteers to deliver the telephone friendship service. In order to match supply of service provision to demand for telephone befriending from participants, the research team contracted the service provider to recruit and train 50 volunteers over a 12-month window between March 2012 and February 2013. This number was intended to retain enough trained volunteers to facilitate twenty 12-week friendship groups for 124 participants between August 2012 and December 2013, notwithstanding unplanned absences and the high levels of volunteer attrition predicted by the literature [36,37]. Because each four-session group facilitation training programme required a minimum of four people and cost £840, it was deemed particularly important that places were filled to ensure capacity for service delivery and efficient use of a finite training budget.

### Randomisation

The randomisation sequence was generated in advance by a CTRU statistician who was not a member of the trial team, without stratification but using blocked randomization with randomly-selected block sizes.

### Blinding

Neither participants nor outcome assessors were blind to treatment allocation. Trial statisticians and the chief investigator were blinded to the treatment allocation codes until after the final analysis. Data presented to the Trial Steering Committee and Trial Management Group did not identify treatment allocations.

### Statistical methods

The early closure of the trial (see below) meant there was no opportunity to follow-up participants recruited from 1 October 2012. As participants randomised to the intervention arm from October 2012 onwards were unable to receive the intervention due to service provider capacity, the primary analysis used an ‘intention-to-treat’ data set. This included all participants randomised before that time plus one participant randomised thereafter who received the intervention due to another participant dropping out prior to receiving the intervention. The ‘per protocol’ data set contained all participants in the control group and participants in the intervention group who completed nine (75%) or more of the group telephone calls over the 12 weeks of group intervention.

As a pilot study, the analysis was largely descriptive and focused on confidence interval estimation and not formal hypothesis testing. Since the intervention is volunteer-led, we also used the data to estimate the ICC.

**Figure 1:**
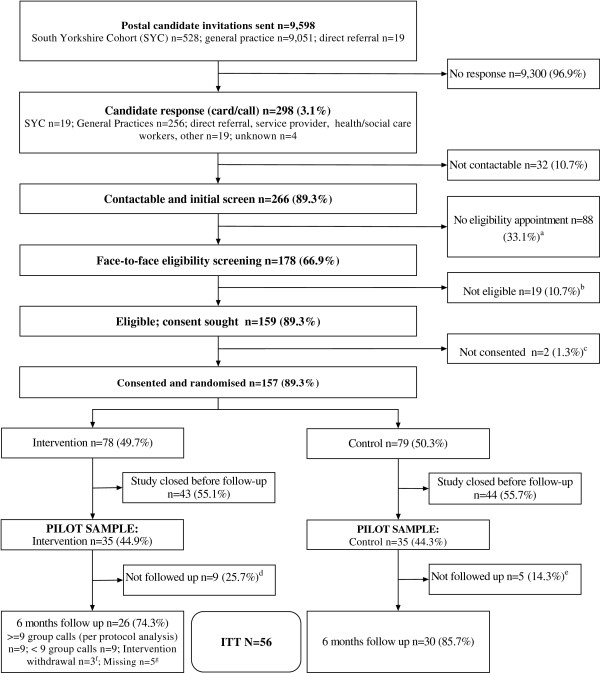
**Flow of participants through the study. **^a^One withdrawn by Chief Investigator due to protocol violation relating to eligibility; two withdrew consent shortly after allocation (one unhappy with involvement of service provider; one felt the study was not for them); five withdrew consent at the point of arranging 6 month follow-up (one due to ill health; one no longer unhappy so did not want to take part; one unhappy with the intervention - at this point they had not received any calls; one other reason - unhappy with being left uninformed about lack of intervention; one due to personal or family issues); one not contactable (minimum of six telephone attempts and reminder letter sent). ^b^One withdrew consent at the point of arranging 6 month follow-up (unhappy with allocated study arm); one not contactable (line dead, letter and email reminder sent); one on 4 week holiday; two refused (one felt no different so did not want to answer the same questions again; one was too ill). ^c^Two participants no longer wanted intervention (one was too busy; one thought intervention was not for them) and one participant did not give any reason for intervention withdrawal. ^d^Assigned to intervention group 4 but the volunteer dropped out before intervention delivery. ^e^One withdrew consent at the point of arranging 6 months follow-up (unhappy with study arm allocation); one not contactable (no dial tone, letter and email reminder sent); one on four week holiday; two refused (one felt no different so did not want to answer the same questions again; one felt too ill). ^f^Two no longer wanted intervention (one was too busy; one thought intervention was not for them); one did not give any reason for intervention withdrawal. ^g^Assigned to intervention group 4 but intervention not delivered as volunteer dropped out. ITT, intention-to-treat.

Baseline and socio-demographic characteristics were summarised and assessed for comparability between trial arms without formal testing of statistical significance [38,39]. Data completeness, based on the primary outcome, is displayed in Figure 1.

We used a marginal general linear model (GLM) with robust standard errors, and an exchangeable correlation to compare the mean SF-36 MH scores from the treatment and control groups [40]. The GLM used Generalised Estimating Equations to estimate regression coefficients. The exchangeable correlation assumes individual outcomes in the same cluster (telephone friendship group) have the same correlation. Participants in the control group were treated as a cluster of size one in the analysis. A 95% CI for the between-arm difference in scores is reported. An adjusted analysis was also performed which included baseline covariates, such as age, sex and baseline SF-36 MH dimension score in the marginal GLM.

Secondary outcomes were analysed in the same way. Estimates of the critical parameters which would be used for a sample size calculation (SD, correlation between baseline and 6-month outcomes and the ICC) are reported.

### Ethical approval

This study received ethics approval from South Yorkshire Research Ethics Committee.

## Results

### Participant recruitment and retention

The team received 298 inquiries about involvement in the trial, of which 275 (92%) were derived from postal invitation and 23 (8%) from other sources (see Figure 1). The first study progression target was met on 30 September 2012 by which time 70 participants had been randomised in the 95 days since recruitment began. As a result, recruitment continued and, between 28 June and 11 January 2012, a total of 157 participants were randomised. By the end of March 2013, the team had successfully followed up 80% (56/70) of the participants randomised before October 2012.

### Delivery of the intervention

Between 17 May 2012 and 22 October 2012, the service provider received 42 expressions of interest from potential volunteers as a result of their advertising campaign and direct approaches. Thirty, however, did not follow up their initial expression of interest to commit to training for the role and, of the twelve who did commence training, only four commenced service provision. One of the four then dropped out due to ill health before delivering the group intervention. The eight who commenced training and then dropped out did so because they could not prioritise volunteering over other commitments including return to employment and fulltime education (n = 3), objected to the allocation of public money to the research team (n = 2), lacked confidence (n = 1), and grew tired of waiting for participant recruitment to start (n = 1). Reasons for discontinuation were not available from one volunteer. Over the same period, four 4-session group training programmes intended for 20 volunteers were conducted, but only 11 volunteers were trained. Lack of take-up meant that three further training programmes, intended to train 15 volunteers, were cancelled between August 2012 and January 2013. The number of days volunteers were retained by the service provider ranged from 12 days to 118 days (mean 62) between point of completing group training and the day they dropped out.

After up to 6 weeks of one-to-one befriending, three volunteers facilitated four 12-week groups (n = 24) between September 2012 and May 2013. However, one group received one-to-one befriending and group facilitation from different volunteers, due to the volunteer attrition described above. As a result of poor recruitment and retention of volunteers, by 17 January 2013, 55 out of 78 participants randomly allocated to the research arm had not been allocated a volunteer facilitator - that is, could not be treated per protocol. For these reasons, and after ruling out the availability of an alternative service provider, the Trial Management Group suspended recruitment to the main trial and made recommendations to the Trial Steering Committee. Shortly after, the funder recommended that the main trial should be terminated when 6-month follow-up was complete for the pilot cohort. These recommendations were accepted.

### Participant characteristics

Tables 1 and 2 show the baseline demographic characteristics and participant reported outcome (PRO) scores for the SF-36, EQ-5D, PHQ-9, de Jong Gierveld scale, ONS well-being outcome and the general self-efficacy scale of the 70 subjects who were part of the internal pilot study. Overall, the two randomised groups were well matched with respect to baseline demographic characteristics.

**Table 1:** Participant baseline characteristics by randomised group (N = 70)

		**Group**					
		**Intervention**			**Control**		
		**N**	**%**		**N**	**%**	
Gender	Female	23	66%		18	51%	
	Male	12	34%		17	49%	
	Total	35	100%		35	100%	
Ethnic group	White European	35	100%		35	100%	
Live with others	No	25	71%		27	77%	
	Yes	10	29%		8	23%	
	Total	35	100%		35	100%	
Main activity	Retired	34	97%		35	100%	
	Looking after home/family	1	3%		0	0%	
	Total	35	100%		35	100%	
Occupation type	Professional	13	38%		8	23%	
	Managerial/Technical	10	29%		10	29%	
	Skilled (non-manual)	1	3%		6	17%	
	Skilled (manual)	3	9%		3	9%	
	Partly skilled	3	9%		4	11%	
	Unskilled	4	12%		4	11%	
	Total	34	100%		35	100%	
		**N**	**Mean**	**SD**	**N**	**Mean**	**SD**
Age (years)		35	81.8	5.8	35	80.1	3.7

Occupation type was left blank by one participant (intervention group) as it was not applicable. They stated main activity as ‘Looking after home/family’).

**Table 2:** Mean baseline participant reported outcomes by randomised group (N = 70)

	**Group**					
	**Intervention**			**Control**		
**Participant reported outcome measure**	**N**	**Mean**	**SD**	**N**	**Mean**	**SD**
SF-36 Physical function	35	65.6	27.4	35	67	27.3
SF-36 Role physical	35	71.3	25.2	35	73.6	25.3
SF-36 Bodily pain	35	64.4	29	35	64	26
SF-36 General health	35	69.2	21.4	35	60	19.4
SF-36 Vitality	35	62.3	20.3	35	54.3	21.4
SF-36 Social function	35	85	22.6	35	81.4	26
SF-36 Role emotional	35	88.6	19.2	35	86.4	24
SF-36 Mental health	35	77.9	17.5	35	74.7	21.6
SF-36 Physical component summary	35	43.8	10.5	35	43.7	11
SF-36 Mental component summary	35	54.1	9.1	35	51.3	12.5
EQ-5D	35	0.73	0.29	35	0.73	0.24
EQ-5D VAS	35	75.1	18.6	35	72.5	18.8
De Jong emotional loneliness	34	1.9	1.8	35	2.3	2
De Jong social loneliness	35	1.4	1.7	35	1.7	1.8
De Jong overall loneliness	34	3.3	3.1	35	4	3.5
PHQ-9	35	2.9	3.6	35	3.3	4.8
ONS wellbeing	35	7.8	2.4	35	7.5	2.5
GSE	35	33.7	4.5	35	31.3	5.5

The Short Form (36) Health Instrument (SF-36) Dimensions are scored on a 0 (poor) to 100 (good) health scale, except for the Physical and Mental Component summary scores which are standardised to have a mean of 50 and SD of 10. The EuroQol 5-Dimension (EQ-5D) utility score is measured on a -0.56 to 1.00 (good health) scale. The EQ-5D visual analogue scale (VAS) is measured on a 0 (worst imaginable health state) to 100 (best imaginable health state). The emotional loneliness scale of the De Jong is scored on a 0 to 6 scale with higher scores indicating more loneliness. The social loneliness scale of the De Jong is scored on a 0 to 5 scale with higher scores indicating more loneliness. The total loneliness scale of the De Jong is scored on a 0 to 11 scale with higher scores indicating more loneliness. The Patient Health Questionnaire (PHQ)-9 is measured on a 0 to 27 scale with higher scores indicating more severe depressive symptoms. General Self-Efficacy (GSE) Scale is scored on a 10 to 40 scale with higher scores indicating more perceived self-efficacy.

The Office for National Statistics (ONS) instrument measures subjective well-being on a 0 to 40 scale, with higher scores indicating high subjective well-being.

One participant did not answer questions 5, 9 and 10 of the De Jong (reason recorded: participant did not want to answer) which affected the emotional and overall loneliness scores.

### Participant reported outcomes

**Figure 2:**
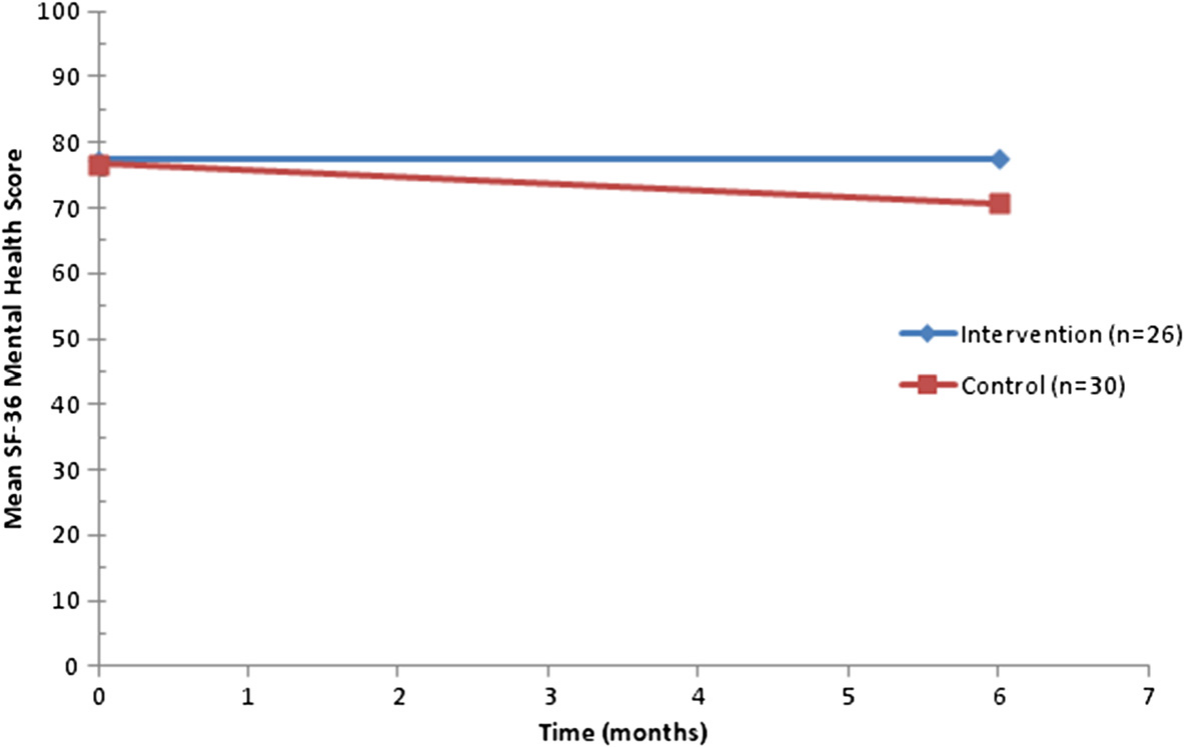
Mean Short Form (36) (SF-36) Mental Health Dimension scores over time by randomised group.

By 6 months post-randomisation follow-up, 56 participants had valid primary outcome data (SF-36 MH Dimension) - 26 in the intervention group and 30 in the control group (Figure 2). The mean SF-36 MH score at 6 months post-randomisation was 77.5 (SD 18.4) in the intervention group and 70.7 (SD 21.2) in the control group, a mean difference of 6.5 (95% CI, -3.0 to 16.0) or 9.5 (4.5 to 14.5), adjusting for age, sex and baseline score (Table 3).

**Table 3:** Mean 6 month post-randomisation follow-up participant reported outcomes by randomised group (N = 56)

	**Group**											
	**Intervention**			**Control**								
**Six-month outcome**	**N**	**Mean**	**SD**	**N**	**Mean**	**SD**	Difference*	**Lower**	**Upper**	Adjusted difference**	**Lower**	**Upper**
SF-36 Mental health	26	77.5	18.4	30	70.7	21.2	6.5	-3	16	9.5	4.5	14.5
SF-36 Physical function	26	60.3	29.9	30	56	29.9	3.4	-10.8	17.5	5	-0.9	10.9
SF-36 Role physical	26	72.6	24.7	30	55.4	27.6	15.6	3.8	27.4	20.2	9.9	30.6
SF-36 Bodily pain	26	71	26	30	53.9	29.8	17.1	2.5,	31.7	16.6	8	25.3
SF-36 General health	26	66.2	24.2	30	56.1	22.9	10.3	-1.2	21.9	2.5	-6.4	11.4
SF-36 Vitality	26	59.4	19.8	30	49.6	25.5	9.8	-2	21.7	3.1	-2.8	9
SF-36 Social function	26	84.1	22.8	30	70	31.1	13.4	1.4	25.4	18.1	7.9	28.3
SF-36 Role emotional	26	89.1	19.4	30	81.7	23.9	7.4	-3.1	17.9	8.6	-0.8	18
SF-36 Physical component summary	26	43.5	10.9	30	38.3	11.5	5.1	-0.4	10.7	4.5	1.4	7.5
SF-36 Mental component summary	26	53.9	9.8	30	49.7	11.5	4.1	-0.5	8.7	4.7	2	7.5
EQ-5D	26	0.73	0.35	29	0.71	0.27	-0.04	-0.17	0.1	0.02	-0.05	0.09
EQ-5D VAS	26	75.5	19.5	30	70.5	21.8	4.7	-4.6	14	5.1	-4.9	15.2
De Jong emotional loneliness	26	2.2	2	30	2.2	1.9	0.2	-0.5	0.9	0	-0.6	0.6
De Jong social loneliness	25	1.3	1.9	30	1.2	1.5	-0.1	-0.7	0.5	0.3	-0.2	0.8
De Jong overall loneliness	26	3.5	3.4	30	3.3	2.9	0	-1	1	0.6	-0.4	1.6
PHQ-9 (6 months)	26	3.1	4	30	3.6	4.6	-0.4	-2.2	1.3	-1.3	-2.6	0
ONS wellbeing	26	8	1.5	30	7.6	1.8	0.5	-0.2	1.2	0.8	0.2	1.4
GSE	26	32.9	4.7	30	32.1	3.8	0.8	-1.5	3.2	1.2	-0.7	3.1

The Short Form (36) Health Instrument (SF-36) Dimensions are scored on a 0 (poor) to 100 (good) health scale. The EuroQol 5-Dimension (EQ-5D) utility score is measured on a -0.56 to 1.00 (good health) scale, except for the Physical and Mental Component summary scores which are standardised to have a mean of 50 and a SD of 10. The EQ-5D visual analogue scale (VAS) is measured on a 0 (worst imaginable health state) to 100 (best imaginable health state). The emotional loneliness scale of the De Jong is scored on a 0 to 6 scale with higher scores indicating more loneliness. The social loneliness scale of the De Jong is scored on a 0 to 5 scale with higher scores indicating more loneliness. The total loneliness scale of the De Jong is scored on a 0 to 11 scale with higher scores indicating more loneliness. The Patient Health Questionnaire (PHQ)-9 is measured on a 0 to 27 scale with higher scores indicating more severe depressive symptoms. General Self-Efficacy (GSE) Scale is scored on a 10 to 40 scale with higher scores indicating more perceived self-efficacy. The Office for National Statistics (ONS) instrument measures subjective well-being on a 0 to 40 scale, with higher scores indicating high subjective well-being.

Models are general linear mixed model with befriending group included as a random effect. *Unadjusted: fixed covariate is randomised group only. **Adjusted: fixed covariates are randomised group, baseline score, age and gender.

Table 3 also shows that, for the secondary PROs such as the other dimensions of the SF-36, the differences in quality of life favoured the intervention group. For five dimensions (role physical, bodily pain, social functioning, physical component summary and mental component summary) after adjustment for baseline score, age and sex, the confidence interval excluded zero, suggesting a non-zero effect. There were no differences in mean scores between the intervention and control groups, observed for the other PROs except for the ONS wellbeing total score.

**Figure 3:**
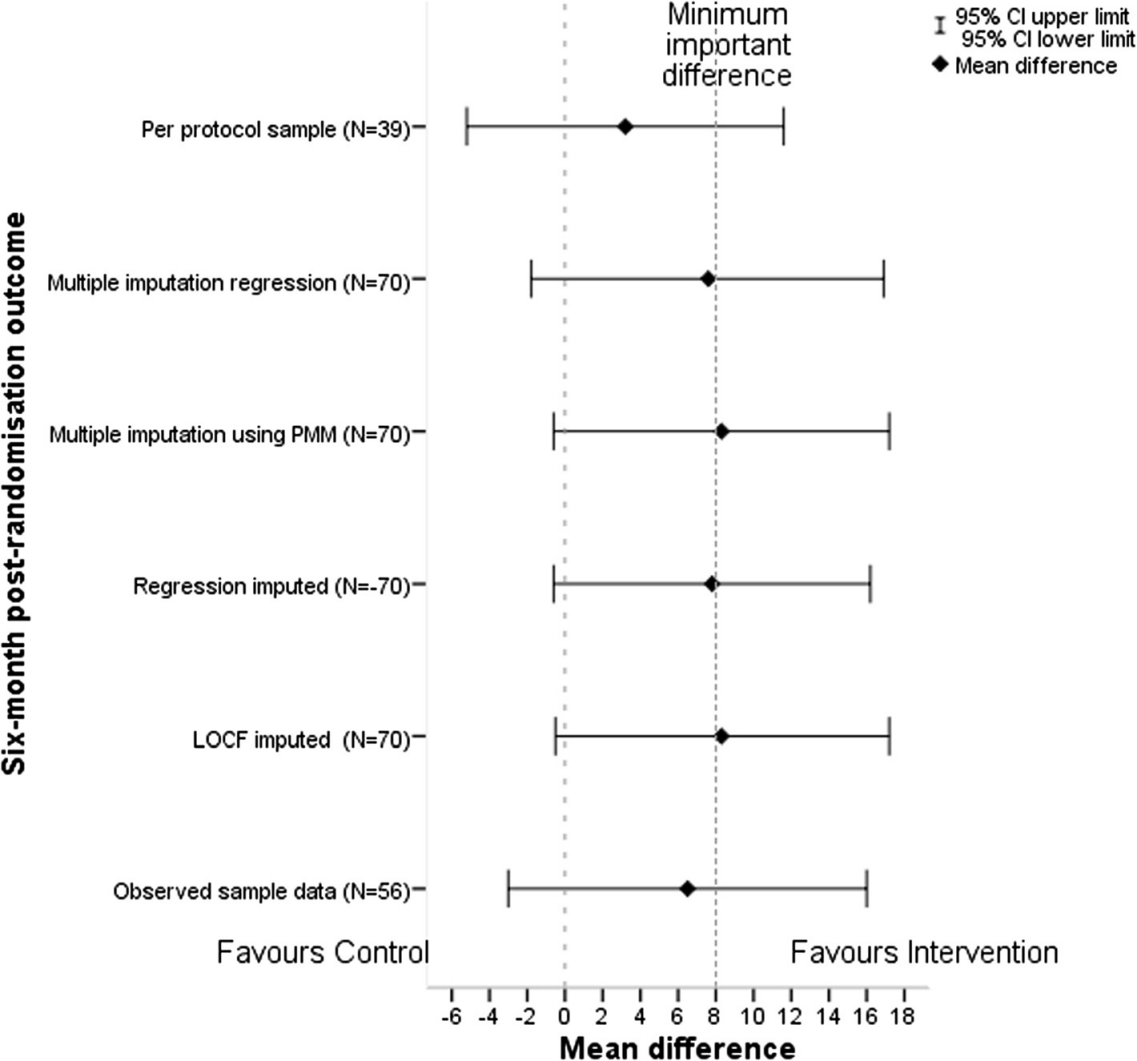
**Forest plot of sensitivity analysis of mean difference in Short Form (36) Health Instrument (SF-36) mental health outcome between groups.** LOCF, last observation carried forward; PMM, predictive mean matching.

The results for the primary outcome were robust to missing data in sensitivity analyses, with all imputation methods producing similar results (Table 4 and Figure 3). Only 35% (9/26) of intervention group participants who had valid 6-month outcome data completed 75% or more of the group intervention telephone calls and were entered in the per-protocol analysis. Six months after randomisation, there was a mean difference in the SF-36 MH score of 3.2 (95% CI, -5.2 to 11.6), or 8.0 (3.3 to 12.7) after adjustment for age, sex and baseline score.

**Table 4:** Mean observed and imputed 6-month post-randomisation follow-up SF-36 MH outcomes by randomised group (N = 70)

	**Group**											
	**Intervention**			**Control**								
**6-month SF-36 MH outcome**	**N**	**Mean**	SD*	**N**	**Mean**	SD*	**Unadjusted difference**	**Lower**	**Upper**	Adjusted difference**	**Lower**	**Upper**
Observed data (N = 56)	26	77.5	18.4	30	70.7	21.2	6.5	-3.0	16.0	9.5	4.5	14.5
LOCF imputed data (N = 70))	35	78	16.6	35	69.6	22.5	8.3	-0.5	17.2	7.7	3.7	11.8
Regression imputed data (N = 70)	35	77.8	16.2	35	69.8	21.4	7.8	-0.6	16.2	7.6	3.6	11.6
Multiple imputation PMM (N = 70)	35	78.9	3.7	35	70.6	3.7	8.3	-0.6	17.2	8.0	2.8	13.3
Multiple imputation regression (N = 70)	35	77.3	3.2	35	69.7	3.9	7.6	-1.8	16.9	7.4	1.8	13.0
Per protocol data (N = 39)	9	73.9	17.5	30	70.7	21.2	3.2	-5.2	11.6	8.0	3.3	12.7

The Short Form (36) Health Instrument Mental Health Dimension (SF-36 MH) is scored on a 0 (poor) to 100 (good) health scale. All analyses use a marginal general linear model, with regression coefficients estimated using generalised estimating equations, with robust standard errors. Regression imputation based on a model with age sex and baseline Mental Health score. Multiple imputation based on 20 imputed data sets, with age, sex and baseline score as covariates, using predictive mean matching (PMM) or linear regression. *For the multiple imputation methods the SD is the standard error of the mean. **Adjusted for randomised group, age, sex and baseline score. *LOCF*, last observation carried forward.

The estimated ICC from the marginal model for the primary outcome was -0.06 indicating little if any clustering by facilitator; the correlation between baseline and 6 months MH scores was 0.78.

## Discussion

This internal pilot trial demonstrated that the trial team could recruit 0.74 participants per day over a 3-month period and collect valid primary outcome data for 80% of randomised participants, rates which fulfilled pre-specified success criteria. It also established that local charitable providers do not necessarily have the resources to match service demand that exceeds what they would usually respond to. The trial was closed early when targets for recruitment and training of volunteer interventionists, built into the subcontract between the University and the service provider, were unmet, leaving 55/78 (70%) of participants randomised to the intervention unable to receive it more than 3 months after randomisation.

The adjusted, between-arm mean difference (9.5; 95% CI, 4.5 to 14.5 points) in the primary outcome (SF-36 MH score at 6 months) is within the 5 to 10 point range defined by the instrument developers as “clinically and socially relevant” [34], but must be treated with caution. The lack of statistical power, the small number of intervention arm participants who received the intervention per protocol, the method of recruitment which was reliant upon individuals opting in and the possibility of resentful demoralisation [41] of control arm participants not blinded to their allocation, all make this estimate of effect problematic for decision-making purposes.

The participant consent rate was 1.6% of those approached about the study, which is in line with rates achieved in other studies evaluating preventive interventions in middle-aged and older populations within the region [14,42]. The same studies also confirm that opt-in recruitment for the purposes of research studies using targeted mail-outs is more reliable than direct referral by health and social care professionals or third sector agencies, if potentially less externally valid. Levels of participant self-efficacy that are high and levels of loneliness that are low, when compared with population norms [43,44], and low uptake rate presents the possibility of participant bias, where the target population for preventive services does not get involved in the RCTs which evaluate them [45,46]. The 20% attrition rate is at the margin of acceptability [47]; comorbidity, exhaustion, and respondent burden frequently result in rates of 20% and 30% in RCTs evaluating health promotion interventions in community-dwelling older people [48].

A strength of our study is that volunteers received standardised training and delivered an intervention that is manualised and therefore more reproducible than most interventions intended to ameliorate social isolation or loneliness [49]. In line with the Medical Research Council Framework [17], a process evaluation, nested within the trial to assess intervention fidelity and quality of implementation, will be published in the NIHR monograph series. The problems experienced by the service provider with the recruitment and retention of volunteers are well-documented in research papers on volunteer management. The retention of volunteers can be affected by personal and organisational factors. The evidence that demographic and psychometric variables are associated with volunteer adherence to work programmes is often weak or contradictory; the factors most strongly associated with retention are educational achievement, prior voluntary experience and life-course stability [36,37]. Policies which require recipients of state benefits to be available for paid work, or which sanction participation by those perceived as ‘workshy’ are thought by some researchers to be creating barriers to sustained volunteering [50]. Two volunteers dropped out of our programme due to pressure to take paid work. Organisational factors are often cited as drivers of volunteer attrition [36,51,52]. Sufficient support to ensure that volunteers are comfortable with their role and its procedures [36,52-55], with a professional volunteer co-ordinator dedicated wholly to the programme, is essential [16]. Volunteers also frequently cite ongoing training as a motivation for programme adherence [36,53,54,56]. More generally, congruence between the goals and ideals of the volunteers and those of the voluntary sector organisations for which they work are thought to promote adherence [36]. The only trial of which we are aware which documented recruitment and attrition of volunteers to deliver a befriending intervention recorded 60/124 (48%) of those expressing interest completing training and 49/60 (82% of those trained) delivering the intervention, compared to 11/42 (26%) and 3/11 (27%), respectively, in our study [57]. Reasons for volunteer attrition were not reported. Unlike in our study, volunteers were recruited and hosted by more than one type of organisation, and resource was available to employ dedicated volunteer co-ordinators, jointly managed by the ‘host’ organisations and the research team. Those wishing to recruit rapidly and retain large numbers of volunteers to telephone befriending programmes should consider using either charitable providers in multiple population centres or outsourcing the work of volunteer recruitment and management to commercial or (where available) state providers.

Our trial adds to tentative evidence that community befriending interventions may be effective in the preservation of good mental health. One systematic review found that, compared with usual care or no treatment, befriending demonstrated small but significant effects on self-reported symptoms of depression in nine studies, five of which followed up for 12 months or more [58]. However, the results should be interpreted with caution as the review authors acknowledged the possibility of publication bias in their work, and only half of the included studies evaluated befriending by lay volunteers, as in our trial. A second systematic review evaluated interventions to reduce loneliness and social isolation in older people [59], including two randomised evaluations of telephone interventions [16,60], and one of a combination one-to-one/group programme like that evaluated in this paper [61]. Whilst the quality of most of the included studies was poor, the review concluded that effective interventions had a theoretical basis and offered “social activity and/or support within a group format” [59]. A third systematic review synthesised the results of RCTs evaluating four strategies to reduce loneliness and social isolation [62]. The conclusion drawn was that social cognitive training interventions yielded greater effect sizes than trials of interventions to enhance social support, improve social skills, or increase opportunities for social interaction, and is unlikely to have applicability to volunteer-led interventions. Its formal analysis, showing that a group-based format was not found to be an effective modifier, is likely to be an artefact of the number of professionally-delivered one-to-one interventions included in the review.

The need for well-conducted studies evaluating theoretically informed, manualised interventions to alleviate loneliness and reduce social isolation in older people remains [49]. Services commissioned especially for research studies are likely to encounter the same issues with matching service supply to demand as demonstrated through our study [15]. The natural tendency of many local voluntary sector organisations may be to deliberately regulate demand for their services based on the resources available to them, resulting in ‘trickle’ recruitment of both volunteers and clients based on self-referral [63]. Ideally services with well-established and effective processes for volunteer recruitment and management systems should be identified to ensure the feasibility of large-scale evaluation. There may also be an argument for non-randomised evaluations if there are significant concerns that trial participants are systematically different from those who would take up the offer of an intervention outside the context of a randomised trial.

## Conclusions

It is feasible to recruit and retain participants aged 75 years or more for an RCT evaluating a volunteer-led telephone friendship group intervention for the maintenance of good mental health. Failure to deliver the intervention at scale led to the early termination of the trial. Future studies should focus on theoretically-based, manualised interventions delivered by service providers with track record of rapidly recruiting and managing large numbers of volunteers. Commissioners of volunteer-led services should be aware that, even where part-time, paid volunteer co-ordinators exist, volunteer recruitment and retention represent a significant management challenge, and such services cannot always be scaled up quickly.

## Abbreviations

6CIT: Six Cognitive Impairment Test; EQ-5D: EuroQol 5-Dimension; GLM: general linear model; GSE: General Perceived Self Efficacy; HRQoL: health-related quality of life; ICC: intra-cluster correlation; MH: Mental Health; NICE: National Institute for Health and Clinical Excellence; NIHR: National Institute for Health Research; ONS: Office for National Statistics; PHQ: Patient Health Questionnaire; PRO: participant reported outcome; RCT: randomised controlled trial; SF-36: Short Form (36) Health Instrument.

## Competing interests

Community Network is a national charity and social enterprise which runs telephone friendship groups and a commercial teleconferencing service for the third sector which could be perceived as having influenced contributions to the report.

## Authors’ contributions

GAM was the chief investigator and conceived of the study. GAM, DH, SJW and RG-W drafted the manuscript. GAM, SJW, DH, AB, MC, CC, RTE and ECG designed the study. RG-W was the study co-coordinator and, with RD, conducted qualitative research interviews. The trial management group were GAM, RG-W, SJW, DH, AB, MC, CC, RTE, AC, CJ and ECG together with Julia Howe of the Sheffield Expert Elder Network, James Goodwin of Age UK, and Kath Horner of NHS Sheffield. The trial management group together with RD and LN and Lauren O’Hara (Sheffield CTRU) helped to implement the study on a day-to-day basis. SJW, supported by Alex Hayman and Ellen Lee, was the study statistician and contributed to the statistical analysis plan and undertook (with SR) the statistical analysis. All authors read, commented on and approved the final manuscript.

## Supplementary Material

Additional file 1 CONSORT 2010 Checklist. Click here for file
